# Daratumumab induces cell-mediated cytotoxicity of primary effusion lymphoma and is active against refractory disease

**DOI:** 10.1080/2162402X.2022.2163784

**Published:** 2023-01-07

**Authors:** Prabha Shrestha, Yana Astter, David A. Davis, Ting Zhou, Constance M. Yuan, Ramya Ramaswami, Hao-Wei Wang, Kathryn Lurain, Robert Yarchoan

**Affiliations:** a HIV and AIDS Malignancy Branch, Center for Cancer Research, National Cancer Institute, Bethesda, MD, USA; b Laboratory of Pathology, Center for Cancer Research, National Cancer Institute, Bethesda, MD, USA

**Keywords:** Daratumumab, primary effusion lymphoma (PEL), pomalidomide, rituximab, all-trans retinoic acid (ATRA), CD38, antibody-dependent cell-mediated cytotoxicity (ADCC), complement-dependent cytotoxicity (CDC), kaposi sarcoma-associated herpesvirus (KSHV), leptomeningeal PEL

## Abstract

Primary effusion lymphoma (PEL), an aggressive non-Hodgkin lymphoma caused by Kaposi sarcoma-associated herpesvirus (KSHV), lacks standard therapy and has a median survival of 10–22 months with combination chemotherapy. PEL is a tumor of plasmablast-like B cells generally expressing CD38, the target of daratumumab (Dara). Initially, we assessed PEL cells from eight patients and established that each expressed high levels of CD38 by flow cytometry. PEL cell lines were also evaluated and most had high CD38 expression. We then assessed Dara’s effects on complement-dependent cytotoxicity (CDC) and antibody-dependent cell-mediated cytotoxicity (ADCC) of PEL cell lines as well as its clinical benefits on two patients with PEL. Despite high CD38 expression, Dara did not induce CDC of PEL cell lines, due in part to high levels of the complement-inhibitory proteins, CD55 and CD59. However, Dara induced significant and dose-dependent increases in ADCC, particularly in those lines with high CD38 levels. Two FDA-approved drugs, all trans-retinoic acid (ATRA) and pomalidomide (Pom), significantly increased surface CD38 levels in low-CD38 expressing PEL cell lines, resulting in increased Dara-induced ADCC. Two patients with refractory PEL were treated with Dara alone or in combination with Pom. One patient with leptomeningeal PEL had a complete response to Dara and Pom combination treatment. Others had improvement in performance status and resolution of malignant ascites with Dara alone. Together, these data support the use of Dara monotherapy or in combination with ATRA or Pom as a potential therapeutic option for PEL.

## Introduction

Primary effusion lymphoma (PEL) is a rare but aggressive non-Hodgkin B-cell lymphoma and occurs primarily in individuals with HIV. It is caused by Kaposi sarcoma-associated herpesvirus (KSHV). KSHV also causes multicentric Castleman disease (MCD), Kaposi sarcoma (KS), and KSHV-associated inflammatory cytokine syndrome (KICS).^1–5^ Approximately 60–90% of PEL tumors are co-infected with Epstein–Barr virus (EBV).^6^ PEL is usually treated with combination anthracycline-containing chemotherapy and antiretroviral therapy (ART). Cures are observed in 40% to 50% of patients, but median overall survival is only about 2 years because many patients experience refractory disease or relapse.^7,8^ Therefore, there is an urgent need to develop new effective therapies for PEL.

PEL cells are derived from post-germinal center B cells and often express plasma-cell markers including CD38, CD138, CD319, and IRF4.^9^ Rituximab, an anti-CD20 monoclonal antibody, has been studied in upfront PEL treatment;^8,9^ however, because PEL generally does not express CD20, any contribution is likely to be indirect, perhaps from killing of other CD20-expressing KSHV-infected cells.

Given that most cases of PEL express CD38,^9^ daratumumab (Dara), a human IgG1 anti-CD38 monoclonal antibody, is a potential treatment candidate. Dara was the first monoclonal antibody to be FDA-approved for use in multiple myeloma (MM) and is being tested in other CD38-positive hematopoietic malignancies. CD38 is expressed at high levels primarily in plasma cell neoplasms and to a certain degree in normal lymphoid and non-hematopoietic cells.^10^ CD38 is a transmembrane glycoprotein with an ectoenzyme function and is involved in NAD^+^ metabolism leading to intracellular calcium homeostasis necessary for cell survival.^10, 11^ CD38 can also bind to CD31 and thereby act as an adhesion molecule or a receptor, although this process is not fully understood.^12,13^

Dara-induced cytotoxicity against MM is mostly mediated by Fc-dependent functions such as complement-dependent cytotoxicity (CDC), antibody-dependent cell-mediated cytotoxicity (ADCC), antibody-dependent cell-mediated phagocytosis (ADCP), and apoptosis via crosslinking.^14–17^ Immune regulatory cells such as regulatory T cells, regulatory B cells, and myeloid-derived suppressor cells also express high CD38 levels;^18–20^ depletion of these cells by Dara can aid in eliminating tumor cells through an increase in CD4^+^ and CD8^+^ T cell number and activity,^20^ which is desirable in patients living with HIV.

In this study, we aimed to assess Dara’s activity against PEL. We assessed CD38 levels on PEL-derived cell lines as well as on PEL cells from eight patients. Using PEL cell lines, we then assessed the ability of Dara to bind to surface CD38 on PEL cells and to induce CDC and ADCC-mediated cytotoxicity. We found that the killing of PEL cells by Dara was mediated by ADCC, but not by CDC, and that variability in CD38 expression on PEL cells contributed to variable Dara responses. We further explored the effects of combining Dara with two other Food and Drug Administration (FDA)-approved drugs: all-trans retinoic acid (ATRA) and pomalidomide (Pom). Finally, we present two case reports demonstrating positive responses following the administration of Dara in patients with refractory PEL.

## Materials and methods

### Cell culture and reagents

PEL cell lines BC-3, BC-1, BC-2, BCBL-1, and JSC-1 were obtained and maintained as described.^21^ Burkitt lymphoma (BL) cell line, Daudi, was obtained from ATCC (Manassas, VA). Jurkat-ADCC bioassay effector cells (Jurkat T-cells with high affinity variant (V158) of human FcγRIIIa and expressing luciferase gene under the control of NFAT response element) used for the reporter assay to measure ADCC induction were obtained from Promega (Madison, WI, cat# G7011). Peripheral blood mononuclear cells (PBMC) were obtained frozen from IQ Biosciences (Berkeley, CA, cat# IQB-PBMC103).

Dara was obtained from Janssen Pharmaceuticals and commercial sources. Pomalidomide (cat# S1567) and rituximab (cat# A2009) were purchased from Selleck Chemical (Houston, TX). All-trans retinoic acid (ATRA) was purchased from Sigma-Aldrich (St. Louis, MO, cat# R2625). Recombinant hIL-2 was obtained from Biolegend (San Diego, CA, cat# 589104). Hyclone super low IgG FBS for use in ADCC reporter bioassay was obtained from VWR (Radnor, PA, cat# 95042–942).

### Flow cytometry for surface expression

**Figure 1.**
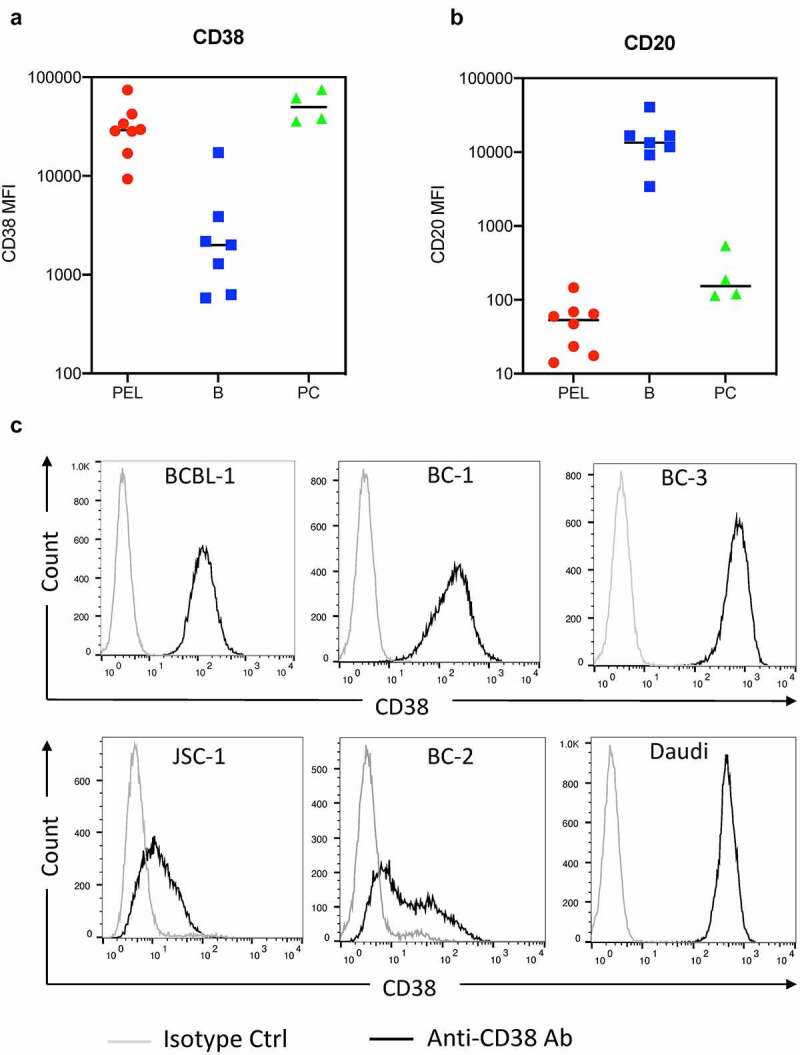
**CD38 and CD20 levels on primary effusion lymphoma (PEL) cells**. (a and b) The levels of surface CD38 and CD20 expression on PEL cells (red), B cells (blue) and plasma cells (green) from effusions (6 pleural fluid and 2 peritoneal fluid) of eight PEL patients were measured by flow cytometry and expressed as median fluorescence intensity (MFI). None of these patients were treated with Dara. The PEL cells were gated by a combination of previously validated light scatter property and antigen expression patterns^26,27^ and without utilizing either CD20 or CD38 gating. One sample did not have detectable B cells while 3 had no detectable plasma cells. (c) Levels of CD38 on the surface of a Burkitt’s lymphoma (BL) cell line, Daudi, and PEL cell lines, BCBL-1, BC-1, BC-3, JSC-1, and BC-2, were measured by flow cytometry using PerCP-Cy5.5 conjugated isotype control or anti-CD38 antibody.

Specimens from PEL patients were processed for multiparametric flow cytometric analysis according to Clinical Laboratory Standards Institute document H43-A2 recommendations.^22^ The procedure included NH_4_Cl red blood cell lysis, phosphate-buffered saline wash, and incubation with a panel of cocktail antibodies. The specimens were acquired on a FACSCanto II flow cytometer (BD Bioscience) and analyzed with FCSExpress software (DeNovo Software, Glendale, CA). PEL cells were detected using a combination of laboratory-developed and validated clinical antibody panels by three hematopathologists (T.Z., C.M.Y and H-W.W.), based on the light scatter property and antigen expression patterns that have been previously characterized.^22,23^ Flow panel used to identify PEL cells from eight patient samples in Figure 1 included antibodies for CD138, CD27, CD28, CD56, CD19, CD20, CD38, and CD45. The phenotype is variable, but the PEL cells can generally be identified by the exhibition of high forward light scatter, expression of some plasma cell markers and dim CD45, and lack of pan-B-cell markers. Flow panel used to identify PEL cells from two Dara-treated patients included antibodies for CD81, CD30, CD43, CD19, CD319, CD14, CD38, and CD45. Gating strategy used to separate B cells and plasma cells and an alternative gating strategy using non-CD38 markers used to identify PEL cells for the determination of CD38 levels are shown in supplemental Figure 1. The geometric mean fluorescence intensity (MFI) of CD38 in all patients was determined by staining with v450-conjugated anti-CD38 antibody (clone HB7, BD Biosciences). Normal hematopoietic cells within the specimens served as internal positive/negative controls.

Levels of various surface markers on PEL and BL cell lines were analyzed by flow cytometry as described previously^23^ using FITC or PerCP/Cy5.5-conjugated antibodies listed in the supplemental method.

### Assessment of Dara binding by flow cytometry

To assess Dara binding to CD38, 5 × 10^5^ cells in 50 µL antibody buffer (PBS with 1% bovine serum albumin [BSA] and 0.05% azide) were incubated with Dara or human IgG1 isotype control antibody (Biolegend, cat# 403502) at indicated concentrations for 30 min at room temperature (RT). Cells were then washed twice with wash buffer (PBS with 10% FBS) and incubated for 1 h in the dark at 4°C with FITC-conjugated IgG anti-human secondary antibody: either rabbit anti-human antibody (cat# SA1-36099) or goat anti-human antibody (cat# 31529) from ThermoFisher at 10 µg/mL or 15 µg/mL final concentration, respectively. Cells were then washed thrice with wash buffer prior to performing flow cytometry analysis. Fold change in median fluorescence intensity (MFI) from Ab-treated cells over background MFI from untreated cells was calculated and data plotted using GraphPad Prism software.

### Complement-dependent cytotoxicity (CDC)

To assess CDC, 10^5^ target cells per 50 µL RPMI media were pre-treated with 50 µL Dara or anti-human IgG isotype control antibody for 15 min at RT prior to incubation with 20% normal human serum (NHS) (Sigma, cat# H4522). After incubation for 2 h at 37°C, propidium iodide (PI) (Sigma) was added at 5 µg/mL final concentration and cell lysis (% of PI-positive cells) analyzed by flow cytometry. To assess CDC after blocking CD55 and/or CD59, 50 µL PEL cells were pre-incubated with 50 µL neutralizing antibody mix (containing isotype matched control antibodies or neutralizing antibodies to either CD55, CD59, or both at 10 µg/mL final concentration each) for 20 min at RT prior to measuring 20% NHS-induced CDC as described above. Neutralizing antibodies used are listed in Supplemental Methods.

### Antibody-dependent cell-mediated cytotoxicity (ADCC) reporter assay

ADCC reporter assay was performed, and data were analyzed using the Jurkat-ADCC reporter bioassay kit (Promega, cat# G7011) according to the manufacturer’s protocol. Briefly, 25 µL target cells (1.5x10^5^) pre-treated with control antibody, Dara, or rituximab were co-incubated with 25 µL Jurkat-ADCC effector cells at a 1:2 or 1:1 effector-to-target ratio for PEL and Daudi cells, respectively, and incubated for 6 h at 37°C before adding 75 µL bio-glo reagent (Promega, cat#G7941). Relative light units (RLU) were measured using Victor X3 multilabel plate reader (PerkinElmer), and luminescence data were plotted using GraphPad Prism software. In ADCC reporter assays performed with PEL cells treated with ATRA or Pom, basal RLU from Jurkat-ADCC cells incubated alone was subtracted prior to calculating fold change in RLU induced by ATRA- or Pom-treatment over that by DMSO control treatment.

### PBMC-mediated ADCC assay

PBMC-mediated ADCC was measured using Calcein-AM (CAM) release assay as described previously.^24^ Frozen PBMCs were rested in RPMI complete media with 50 to 100 U/mL hIL-2 for 18 h prior to their use in ADCC assays. Briefly, PEL cells were stained with 3 µM CAM (ThermoFisher, cat# 65–0853-78) at 5 × 10^5^ cells per mL RPMI media without phenol red (assay buffer) (ThermoFisher, cat# 11835030). 10^4^ CAM-stained cells per 50 µL assay buffer were incubated with 50 µL Dara for 15 min at RT prior to incubation with 100 µL PBMCs at a 100:1 to 50:1 effector-to-target ratio at 37°C for 4 h. Wells with only target cells and those with 2% Triton X-100 were used for spontaneous and maximal calcein release controls, respectively. After 4 h, 100 µL supernatant was transferred to a black plate and relative fluorescence unit (RFU) was measured using Victor X3 multilabel plate reader (PerkinElmer) at excitation/emission wavelengths of 485 nm and 530 nm. Percent lysis was calculated as described previously.^24^ Percent-specific lysis was then calculated by subtracting percent lysis obtained in the absence of Dara.

### Treatment of patients with PEL

We identified two patients with refractory PEL treated with Dara alone or with pomalidomide in the HIV and AIDS Malignancy Branch (HAMB), Center for Cancer Research, National Cancer Institute (NCI). PEL was diagnosed via cytopathology and flow cytometry of effusions or via biopsy of extracavitary masses. All patients were enrolled on an NCI Institutional Review Board-approved protocol for patients with viral infections and/or cancer (NCT00006518). Patients gave written informed consent in accordance with the Declaration of Helsinki.

### Statistical analysis

Statistical analysis was performed using two-tailed student’s *t-test* (paired or unpaired where indicated) in experiments with at least three biological replicates. *P*-values less or equal to 0.05 were considered statistically significant.

## Results

### CD38 expression on PEL cells from patients and PEL-derived cell lines

We first determined the levels of CD38 in PEL samples from eight patients treated in the HAMB. PEL cells were identified based on established parameters for various antigen expression patterns without utilizing either CD20 or CD38 gating.^25,26^ Gating strategy and representative flow cytometric analyses showing the phenotypic profiles of PEL cells, normal B cells, and plasma cells are shown in supplemental Figure 1. All 8 PEL samples expressed very high levels of surface CD38 (Figure 1a) and very low levels of CD20 (Figure 1b). We then analyzed CD38 surface levels on various PEL cell lines by flow cytometry using PerCP-Cy5.5-conjugated anti-CD38 antibody. All the lines expressed CD38, but at variable levels (Figure 1c). Three of the five lines (BCBL-1, BC-1, and BC-3) expressed high CD38, which was comparable to that in Daudi, a BL line (Figure 1c). The other two lines, JSC-1 and BC-2, expressed relatively lower CD38.

### Dara binding to surface CD38 on PEL cell lines

**Figure 2.**
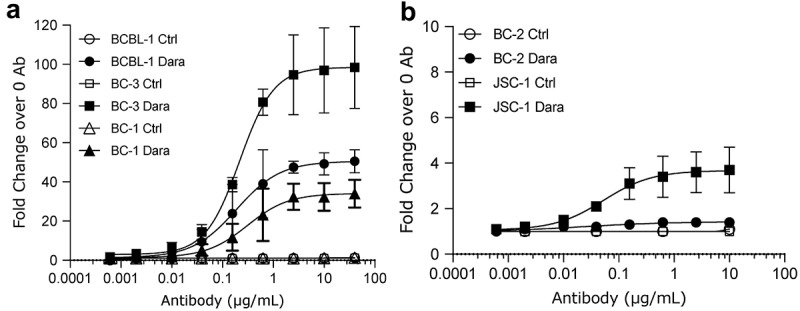
**Binding of Dara to surface CD38 on PEL cell lines**. Binding of various concentrations of Dara to CD38 on the surface of PEL cell lines was measured by flow cytometry. Shown are high-CD38 lines BCBL-1, BC-3, and BC-1 cells (a) and low-CD38 lines BC-2 and JSC-1 (b) stained with various concentrations of Dara or human IgG1 isotype control and then stained with FITC-conjugated anti-human IgG 2° Ab. Data are expressed as fold change in median fluorescence intensity (MFI) from Ab-treated cells over background MFI from untreated cells and shows average and standard deviations from 3 separate experiments in (a) and 2 separate experiments in (b).

The binding of Dara to CD38 on PEL lines was analyzed by flow cytometry using FITC-conjugated anti-human IgG secondary antibody. Dara showed dose-dependent binding to CD38 on PEL cell surfaces, achieving saturation at around 1 µg/mL in high CD38-expressing lines (Figure 2a) and 0.2 µg/mL in low CD38-expressing lines (Figure 2b). These concentrations are well within clinically achievable levels.^27^

### Dara does not induce CDC of PEL cell lines

**Figure 3.**
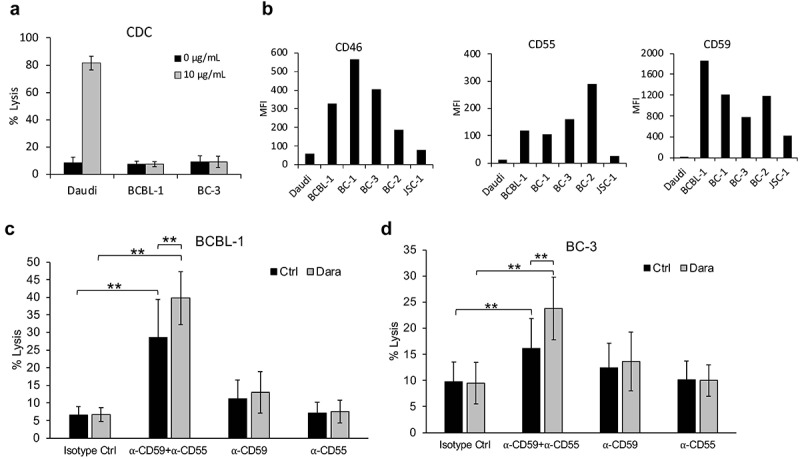
**Lack of Dara-mediated complement-dependent cytotoxicity (CDC) of PEL cell lines**. (a) CDC in the presence of 20% pooled normal human serum was performed on Daudi, a BL cell line, and BCBL-1 and BC-3 PEL lines, in the presence or absence of 10 µg/mL Dara. Cells were stained with propidium iodide (PI) and % lysis was determined based on PI-positive cells using flow cytometry after 2 h. (b) Levels of complement-inhibitory proteins CD46, CD55, and CD59 on the surface of Daudi or various PEL cell lines were measured by flow cytometry after staining the cells with PerCP-Cy5.5-conjugated anti-CD46 or anti-CD55 antibodies or FITC-conjugated anti-CD59 antibody. Data are expressed as median fluorescence intensity (MFI). (c and d) BCBL-1 (c) and BC-3 (d) cells were incubated with 10 µg/mL isotype control or neutralizing antibodies against CD59, CD55, or both prior to performing CDC assays with or without 10 µg/mL Dara as described in (a). Data are presented as % lysis as determined by PI-positive cells using flow cytometry. Error bars represent standard deviations from at least 3 independent experiments. Statistically significant differences (*P ≤ .05, **P ≤ .01) by 2-tailed t-test are indicated.

We analyzed whether Dara could induce CDC of PEL cell lines using 20% NHS as source of complement. While Daudi cells showed a robust CDC (80% lysis) with 10 µg/mL Dara, neither BCBL-1 nor BC-3 PEL lines showed CDC under identical conditions (Figure 3a). Further increasing Dara concentration to 100 µg/mL also failed to induce CDC in all 5 PEL lines (supplemental Figure 2A).

High levels of membrane-bound complement-inhibitory molecules CD46, CD55, and CD59 are associated with resistance to therapeutic mAb in various cancers.^28–32^ The levels of all three of these proteins were higher in PEL lines compared to Daudi although there was some variation among the lines (Figure 3b; supplemental Figures 2B and 2C). CD59, and to some degree CD55, were highly elevated in PEL, so we explored whether blocking CD59 and/or CD55 might reverse the inability of Dara to induce CDC of PEL cell lines. To this end, we blocked CD59 and/or CD55 on the PEL cell surface using neutralizing antibodies prior to assaying CDC. Blocking CD59 or CD55 alone did not increase CDC on either BCBL-1 (Figure 3c) or BC-3 lines (Figure 3d). However, blocking both CD55 and CD59 raised baseline CDC of both cell lines in the absence of Dara (Figure 3c and 3d). CDC of these cells was further elevated upon Dara-treatment (Figure 3c and 3d), although the percent lysis was only approximately 40% and 25% in BCBL-1 and BC-3 cells, respectively, suggesting that high CD55 and CD59 levels only partially account for the resistance of PEL cell lines to Dara-mediated CDC. We were unable to identify an effective antibody against CD46 to assess if blocking this molecule would enhance PEL CDC.

### Dara induces ADCC of PEL cell lines

**Figure 4.**
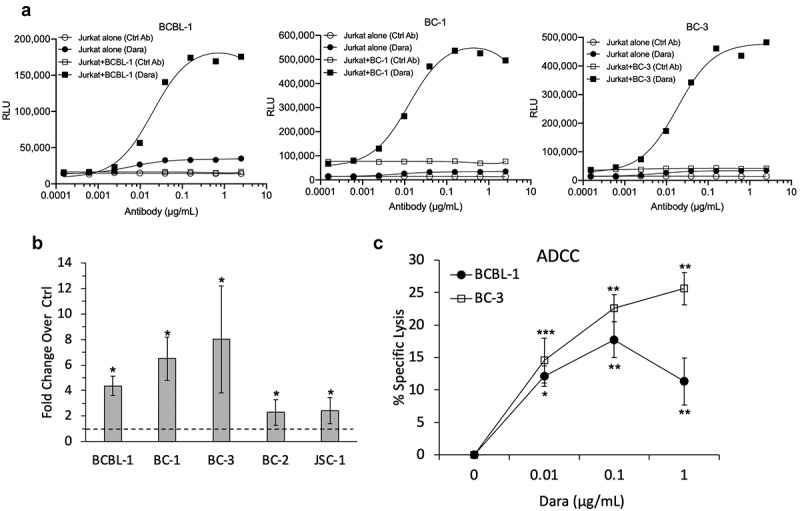
**Dara-induced ADCC of PEL cell lines**. (a) ADCC induction by Dara was assessed using Jurkat-ADCC cells (expressing Fc*Y* receptor and luciferase-driven NFAT response element) as the effector cells. BCBL-1, BC-1, or BC-3 cells were treated with various concentrations of Dara or human IgG isotype control ab and co-incubated with Jurkat-ADCC cells; luciferase activity was measured after 6 h. Data are presented as relative luminescence unit (RLU) from Jurkat-ADCC cells alone or Jurkat-ADCC cells co-incubated with PEL cells in the presence or absence of Dara from one representative experiment. (b) ADCC pathway activation in Jurkat-ADCC cells by 0.6 ug/mL Dara-treated PEL cell lines presented as fold change in luciferase activity over control-treated cells. Data represent an average from 3 independent experiments (4 experiments for BC-3) and error bars represent standard deviations. (c) PBMC-mediated ADCC of BCBL-1 and BC-3 cells as measured by calcein-AM release assay. Percent specific lysis was calculated by subtracting the % lysis in the absence of Dara from that in its presence. Data represent an average of 4 (BCBL-1) or 3 (BC-3) separate experiments and error bars represent standard deviations. Statistically significant differences (*P ≤ .05, **P ≤ .01, ***P ≤ .001) relative to controls without Dara as calculated using 2-tailed t-test are indicated.

We next assessed whether Dara could induce ADCC of PEL cell lines. Using Jurkat-ADCC effector cell-based luciferase assay, we found that PEL cell lines induced dose-dependent increases of ADCC pathway activation in the presence of Dara but not in the presence of isotype control human IgG antibody (Figure 4a). ADCC induction by high-CD38 expressing cells ranged from approximately four- to eight-fold in the presence of 0.6 µg/mL Dara compared to controls (Figure 4b). By contrast, low-CD38 expressing BC-2 or JSC-1 cells yielded significant but lower ADCC induction (~2-fold) (Figure 4b).

Next, the ability of Dara to induce ADCC of PEL lines mediated by PBMCs from healthy donors was tested using calcein-AM release assay. Dara dose-dependently led to marked and significant increases in specific lysis of both BCBL-1 and BC-3 lines when incubated with PBMCs (Figure 4c). Taken together, these data suggest that Dara can induce ADCC of PEL, particularly those with high CD38 expression.

### Rituximab does not induce CDC or ADCC of PEL cell lines

**Figure 5.**
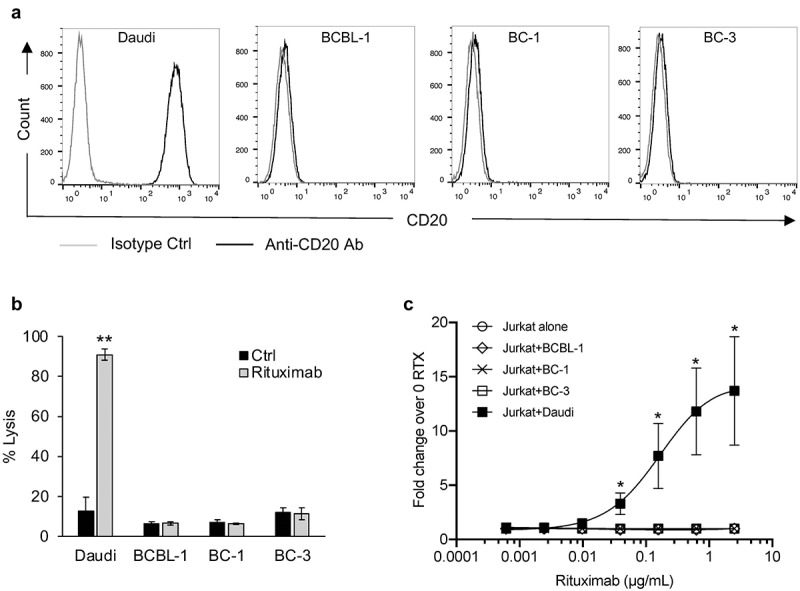
**Rituximab does not induce complement-dependent cytotoxicity (CDC) or antibody-dependent cell-mediated cytotoxicity (ADCC) against PEL cell lines**. (a) Levels of CD20 on the surface of a BL cell line, Daudi, and PEL cell lines, BCBL-1, BC-1, and BC-3, were measured by flow cytometry using PerCP-Cy5.5 conjugated isotype control or anti-CD20 antibody. (b) Complement dependent cytotoxicity (CDC) in the presence of 20% pooled normal human serum was performed on Daudi and PEL cell lines treated with 10 µg/mL rituximab or control ab. Cells were stained with propidium iodide (PI) and % lysis was determined based on PI-positive cells using flow cytometry. (c) ADCC induction by rituximab (RTX) assessed using Jurkat-ADCC cells as the effector cells. Daudi or PEL cell lines were treated with various concentrations of rituximab and co-incubated with Jurkat-ADCC cells and luciferase activity was measured after 6 h. Data are presented as fold change in luciferase activity in the presence of rituximab over that in its absence. Error bars represent standard deviations from 3 independent experiments. Statistically significant differences (*P ≤ .05, **P ≤ .01) by 2-tailed t-test are indicated.

Although PEL generally does not express CD20, rituximab has been administered experimentally as part of a multi-drug regimen^7,8,33,34^ and we explored whether rituximab can lead to CDC or ADCC of PEL cell lines. We first assayed CD20 levels on the surface of PEL cell lines by flow cytometry and confirmed that they are indeed negative for CD20 (Figure 5a). Moreover, rituximab-treated PEL cells failed to show killing by CDC (Figure 5b) or induction of ADCC (Figure 5c). By contrast, Daudi, a BL cell line expressing high level of CD20 (Figure 5a), manifested both activities (Figures 5b and 5c). These results suggest that any beneficial effects of rituximab in PEL are not due to direct killing of the PEL cells through these mechanisms.

### All-trans retinoic acid (ATRA) increases CD38 levels and ADCC induction of PEL cell lines

**Figure 6.**
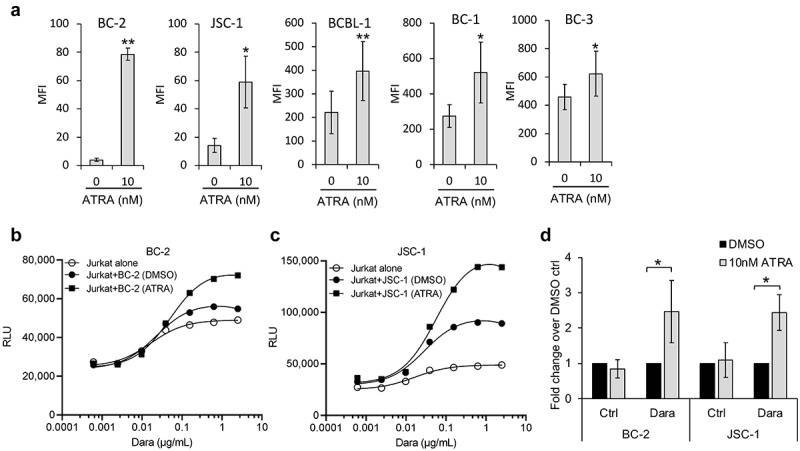
**All-trans retinoic acid (ATRA) increases CD38 expression of PEL cell lines and enhances Dara-mediated ADCC induction**. (a) Levels of surface CD38 on PEL cell lines treated with DMSO alone (0 nM ATRA) or with 10 nM ATRA for 72 h were measured by flow cytometry using PerCP-Cy5.5 conjugated anti-CD38 antibody and expressed as median fluorescence intensity (MFI) after subtracting background MFI from isotype control ab. Results represent average MFIs and standard deviations from at least 3 separate experiments. (b and c) ADCC pathway activation by DMSO or 10 nM ATRA-treated BC-2 (b) and JSC-1 (c) cells after co-incubation with Jurkat-ADCC cells for 6 h in the presence of various concentrations of Dara. Data show one representative experiment from 3 separate experiments and is expressed as relative luminescence unit (RLU) from Jurkat-ADCC cells alone or co-incubated with the PEL lines. (d) Change in ADCC by ATRA-treated BC-2 and JSC-1 cells over that by DMSO-treated cells is presented as fold change in RLU from Jurkat-ADCC cells after 6 h co-incubation in the presence of 0.6 µg/mL DARA or isotype control ab. Data show averages and standard deviations from 4 separate experiments. Statistically significant differences (*P ≤ .05, **P ≤ .01) by 2-tailed t-test are indicated.

All-trans retinoic acid (ATRA) is FDA-approved for the treatment of acute promyelocytic leukemia and raises surface CD38 and decreases CD55 and CD59 levels in Dara-refractory MM cell lines as well as patient-derived cells.^32,35,36^ Therefore, we tested whether ATRA could enhance the cytotoxic activity of Dara against PEL. Treatment of the low CD38-expressing lines, BC-2 and JSC-1, with 2.5 to 40 nM ATRA showed a dose-dependent increase in surface CD38 levels up to around 10 nM ATRA, after which no substantial increase was observed (supplemental Figure 3A). At 10 nM ATRA, all five PEL lines, regardless of basal CD38 levels, showed significant increases in CD38 levels, although the increases were higher in lines with low basal CD38 (Figure 6a; supplemental Figure 3B). Of note, 10 nM ATRA did not cause any effect in cell growth (supplemental Figure 3C) and, unlike MM cells,^32,35^ did not change complement-inhibitory proteins in PEL cell lines (supplemental Figure 4). BC-2 and JSC-1 cells treated with 10 nM ATRA showed significant increases in Dara-induced ADCC induction in Jurkat-ADCC cells relative to control-treated cells (Figures 6b-d). We then attempted to measure PBMC-mediated ADCC of these low-CD38-expressing cell lines using calcein-AM release assay 3 d after their treatment with control or 10 nM ATRA. Interestingly, in control-treated BC-2 and JSC-1 cell lines, Dara led to small decreases in lysis, although the decrease was only significant for 1 µg/mL Dara (supplemental Figures 5A and 5B). In ATRA-treated cells, we observed a small trend toward an increase in lysis in the presence of Dara (supplemental Figures 5C and 5D).

### Pomalidomide increases CD38 levels and ADCC induction of low-expressing PEL cell lines

**Figure 7.**
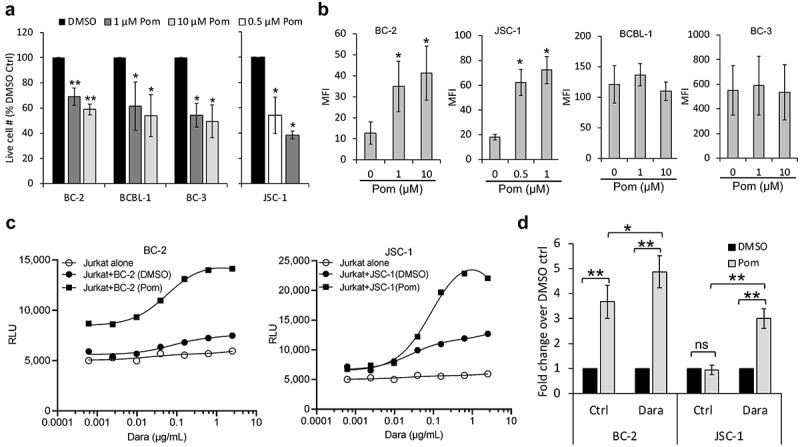
**Pomalidomide increases CD38 expression of PEL cell lines and enhances Dara-mediated ADCC**. (a) PEL cell lines were treated for 72 h with DMSO control (0 Pom) or indicated concentrations of Pom and then the live cell number was counted after trypan blue-staining. Live cell number in Pom-treated cells as a percentage of DMSO-treated cells are presented. (b) Levels of surface CD38 on PEL cell lines treated with DMSO or Pom for 72 h were measured by flow cytometry using PerCP-Cy5.5 conjugated anti-CD38 antibody and expressed as median fluorescence intensity (MFI) after subtracting background MFI from isotype control ab. Results represent average MFIs and standard deviations from at least 3 separate experiments. Because of toxicity at higher concentrations, the highest concentration of Pom utilized in JSC-1 cells was 1 µM. (c) ADCC pathway activation by DMSO or Pom-treated PEL cells (BC-2 with 1 µM and JSC-1 with 0.5 µM Pom) after co-incubation with Jurkat-ADCC cells for 6 h in the presence of various concentrations of Dara. Data show one representative experiment from three separate experiments and is expressed as relative luminescence unit (RLU) from Jurkat-ADCC cells alone or co-incubated with the PEL lines. (d) Change in ADCC-induction by Pom-treated BC-2 and JSC-1 cells over that by DMSO-treated cells is presented as fold change in RLU from Jurkat-ADCC cells after 6 h co-incubation in the presence of 0.6 µg/mL Dara or isotype control ab. Data show averages and standard deviations from 4 separate experiments. Statistically significant differences (*P ≤ .05, **P ≤ .01) by 2-tailed t-test are indicated.

Pomalidomide (Pom), an immunomodulatory drug approved for use with Dara in relapsed MM, has been shown by our group to increase expression of certain immune surface markers in several virus-induced tumor lines.^23,37,38^ Pom also inhibits the growth of PEL lines *in vitro*^37,39^ (Figure 7a). We assessed whether Pom could enhance Dara’s activity against PEL cells by raising CD38 expression. Pom significantly upregulated CD38 levels on low-CD38 expressing PEL lines (BC-2 and JSC-1), but not on high-CD38 expressing lines (BCBL-1 and BC-3) (Figure 7b). Increased CD38 expression by Pom also led to an increase in Dara-induced ADCC as measured by Jurkat-ADCC reporter assay (Figures 7c and 7d). Interestingly, while ADCC induction by Pom-treated JSC-1 cells was higher only in the presence of Dara, ADCC induction by Pom-treated BC-2 cells increased by 3.5-fold even in the absence of Dara (Figure 7d), and it increased further to approximately 4.5-fold over control-treated cells in the presence of Dara (Figure 7d). We then measured PBMC-mediated ADCC of these cell lines using calcein-AM release assay 3 d after their treatment with DMSO control or Pom. Both BC-2 and JSC-1 cells showed a trend toward increased lysis by Dara when treated with Pom compared to control treatment (supplemental Figures 5E and 5F). Taken together, these data suggest that Pom can enhance ADCC activity against PEL cells with low basal surface CD38 levels. By contrast, Pom did not lead to significant changes in surface CD59 (supplemental Figure 6) and both ATRA and Pom did not change CD20 expression on PEL lines (supplemental Figure 7).

### Treatment of PEL with Dara alone or in combination with pomalidomide

We treated two patients with refractory PEL using Dara alone or in combination with Pom. PEL cells from both patients expressed high CD38 levels as demonstrated by flow cytometry performed prior to starting Dara-treatment (supplemental Figure 8).

Case 1: A 37-year-old cisgender man diagnosed with HIV and primary refractory PEL was transferred to NCI in 2019 after 2 prior lines of therapy (supplemental Table 1). Upon transfer, the patient’s Eastern Cooperative Oncology Group (ECOG) performance status was 4, HIV viral load was undetectable, and CD4^+^ T cell count was 143 cells/µL. In addition to extensive abdominal involvement by PEL, cerebrospinal fluid (CSF) evaluation revealed leptomeningeal PEL. He was treated with 200 mg pembrolizumab every 3 weeks, 4 mg Pom daily 21 out of 28 d, and intrathecal methotrexate with improvement in his abdominal symptoms but persistent leptomeningeal disease. In May 2020, his regimen was changed to intravenous Dara 16 mg/kg weekly with subsequent resolution in his abdominal pain and ascites, as well as improvement in ECOG performance status to 1. Due to the COVID-19 pandemic, he was unable to return to the NCI after August 2020, and he died in September 2020 due to progressive PEL.

Case 2: A 53-year-old cisgender man with HIV and a prior history of KS and KSHV-MCD presented in 2017 with a left cervical neck mass found to be extracavitary PEL. CSF evaluation revealed leptomeningeal involvement by PEL. His HIV viral load was undetectable with a CD4^+^ T cell count of 358 cells/µL. While his systemic PEL went into complete remission after front-line combination chemotherapy (see supplemental Table 2), he had refractory leptomeningeal disease. In August 2020, the patient began treatment with subcutaneous Dara 1800 units weekly and Pom 4 mg daily 21 out of 28 d. By December 2020, the patient’s CSF was negative for PEL by flow cytometry and cytology. In January 2021, the patient was hospitalized with severe COVID-19 requiring prolonged intubation, and Dara and Pom were withheld. Upon discharge in April 2021, his CSF was again positive for PEL, and he was restarted on subcutaneous Dara 1800 units weekly. Pom was added to Dara in January 2022 as the CSF remained positive for PEL, but this regimen did not clear the CSF a second time and both agents were discontinued in June 2022.

## Discussion

Our data demonstrate that most PEL cell lines as well as patient-derived PEL cells express surface CD38 although the levels vary among the PEL-derived cell lines. We also show that Dara can induce ADCC-mediated lysis of PEL cell lines but not CDC, and that ATRA or Pom can enhance expression of CD38 and increase the susceptibility of even low-CD38-expressing cells to Dara-induced lysis. We further describe two clinical cases in which refractory PEL responded to treatment with Dara, alone or with Pom.

Rituximab is commonly used to treat a variety of CD20-positive lymphomas.^40^ It has been used along with other agents in the successful treatment of PEL, although its contribution is unclear.^7,8,33,34^ Interestingly, rituximab has been shown to have activity in KSHV-associated MCD even though the KSHV-infected plasmablasts in KSHV-MCD do not express CD20.^41,42^ Here, we provide evidence that rituximab does not lead to CDC or ADCC in PEL lines. This suggests that any benefit rituximab might provide PEL (or KSHV-MCD) is likely to be indirect, potentially by depleting KSHV-infected B cell reservoir, a source of inflammatory cytokines.

PEL generally expresses high levels of CD38, suggesting Dara may be a promising treatment option. Consistent with a recently published report by Panaampon et al.,^43^ we found that Dara can induce ADCC-mediated lysis of PEL cell lines, particularly those with high surface CD38 levels. However, in contrast to that study, we failed to find that Dara could induce CDC in the PEL cell lines tested. This difference may be attributed to our use of human serum as a source of complement as opposed to the rabbit serum used by Panaampon et al.^43^ Complement-inhibitory membrane proteins CD46, CD55, and CD59 are species-specific, inhibiting human but not non-human complement.^44,45^ All PEL cell lines expressed high levels of these proteins compared to Daudi, a BL cell line that readily shows Dara-induced CDC in the presence of human complements. Neutralizing both CD55 and CD59 on the PEL cell surface led to a significant albeit incomplete rescue of Dara-induced CDC of PEL cell lines, suggesting that expression of these proteins contributes to the resistance of PEL to Dara-mediated CDC. Additionally, inhibiting CD46 may further enhance Dara-induced CDC,^46^ although we were unable to test this directly.

We also report two patients with refractory PEL who derived significant benefit from treatment with Dara, including one patient whose primary refractory leptomeningeal PEL had a complete response to the combination of Dara and pomalidomide. Two other cases of PEL treated with Dara have previously been reported.^47,48^ Overall, these results provide evidence that Dara has activity in PEL, including PEL that involves the CSF, which is notoriously difficult to treat. We show that the activity of Dara in PEL is due in part to its ADCC activity, but other direct or indirect activities may contribute to the clinical activity.

While PEL samples from patients and most PEL cell lines have high CD38 levels, some PEL lines express relatively little. Low CD38-expressing lines showed a smaller Dara-induced ADCC, consistent with previous observations in MM and CLL cells where Dara-mediated cytotoxic effects correlated with the surface CD38 levels.^24,35^ Interestingly, we found that ATRA or Pom alone can raise surface CD38 on the PEL cell lines, particularly those with low CD38 levels. Consistent with previous reports in MM cells,^35,49^ ATRA and Pom-induced increase in CD38 was also associated with an enhanced potential of the PEL cell lines to undergo ADCC as measured by Jurkat-ADCC-based reporter assay. Examination of these effects in the calcein-AM release assay was more challenging; while no effect of ATRA was observed, a trend toward an increase was seen in Pom-treated cells. Additional studies may help assess the potential effects of these drugs on Dara-induced killing of low-CD38-expressing PEL cells. Pom, in particular, is of interest in this regard because it has been shown previously to cause apoptosis and cell cycle arrest of PEL cells *in vitro* as well as enhance NK and T-cell targeting of PEL cell lines by increasing expression of immune activating molecules MHCI-1, ICAM-1, and B7-2 on the cell surface.^37–39^ Moreover, Pom has been shown to increase CD38 on regulatory T-cells, thus targeting them for Dara-mediated elimination and enhancing anti-tumor immunity of MM.^50^ Similarly, Dara combined with Pom and dexamethasone yielded clinical response in 33% of lenalidomide-refractory MM patients who were resistant to both Dara and Pom.^51^ These observations and the multifaceted functions of Pom on PEL provide a rationale for considering the use of Pom (or ATRA) in combination with Dara in PEL. The findings from our clinical case also support this approach.

Since ADCC appears to be the primary mechanism of Dara’s activity against PEL cell lines, other therapeutic interventions that enhance ADCC activity might be worth exploring to enhance Dara’s activity in PEL. One such approach is extracorporeal photopheresis (ECP), a leukapheresis-based immunomodulatory procedure that is FDA-approved for use in Sézary syndrome.^52^ Although the exact mechanism of action of ECP is not well understood, recent studies have shown that a positive response to ECP might be mediated at least in part due to enhanced NK cell activity.^53,54^ In addition, newer agents that are currently being developed to specifically enhance NK-activity, such as anti-KIR monoclonal antibodies^55,56^ and off-the-shelf adoptive NK cell immunotherapy,^57^ might also be good candidates for combination use with Dara in PEL. Future *in vitro* as well as *in vivo* experiments with PEL xenograft models should provide a better understanding of the utility of these combination therapies. Overall, data presented here provide evidence to support a clinical trial of Dara in patients with refractory/relapsed PEL and in the future, potentially exploring the combination of Dara with ATRA, Pom, or other ADCC-enhancing therapy.

## Supplementary Material

Supplemental Material

## Data Availability

The data that support the findings of this study are presented in the main text or the supplementary material. Additional data that support the findings of this study are available from the corresponding author [R.Y.], upon reasonable request.
