# Supplementary material for: Daratumumab induces cell-mediated cytotoxicity of primary effusion lymphoma and is active against refractory disease
Source: Oncoimmunology. 2023 Jan 7;12(1):2163784. doi: 10.1080/2162402X.2022.2163784 (PMC9828731; doi:10.1080/2162402X.2022.2163784)
Supplement: Supplemental Material [file KONI_A_2163784_SM0036.pdf]

## Supplemental Methods

### Antibodies used to determine surface expression of various markers on cell lines by flow cytometry

PerCP-Cy5.5-conjugated antibodies used were anti-CD38 (BD Biosciences, cat # 561106), anti-CD46 (cat# 352413), anti-CD55 (cat# 311315), anti-CD20 (cat# 302326), and IgG1 and IgG2b isotype controls (cat #s 400150 and 400338, respectively) from Biolegend. FITC-conjugated antibodies used were anti-CD59 (Biolegend, cat#304706) and its isotype control (Sigma, cat # F6522).

### Blocking antibodies used in CDC

Blocking antibodies used were IgG1 anti-CD55 antibody (BRIC-216) (ThermoFisher, cat# MA1-91161) and IgG2 anti-CD59 antibody (MEM-43) (Novus Biologicals, Littleton, CO, cat# NB500-330). Isotype matched antibodies used were IgG1 control antibody (ThermoFisher, cat# 16-4714-85) and IgG2 control antibody (Novus, cat# MAB003R).

## Supplemental Figure Legends

**Supplemental Figure 1. Flow cytometry showing gating strategy and phenotypic profiles of PEL cells, B cells, and plasma cells from patients.** Representative flow cytometric analyses are shown for cells in the pleural fluid of one PEL patient (A) Flow cytometry analysis showing gating strategy for normal B cells (blue) and plasma cells (green) using antibodies against CD19, CD20, and CD38. (B) Representative flow cytometry showing combinational alternative non-CD38 gating strategy used to identify PEL cells (red circles) for the purpose of CD38 measurement. (C) Representative flow cytometric analysis showing the phenotypic profiles of PEL cells (red), B cells (blue) and plasma cells (green). The PEL cells express CD38 but lacks CD19 and CD20. The normal plasma cells express both CD19 and bright CD38 but are negative for CD20. Normal B cells express CD19, CD20, and variable CD38.

**Supplemental Figure 2. Dara does not lead to CDC of PEL cell lines in part due to high expression of complement-inhibitory proteins.** (A) CDC in the presence of 20% pooled normal human serum was performed on BCBL-1, BC-1, BC-3, BC-2, and JSC-1 PEL cell lines, treated with 0, 1, 10, or 100 μg/mL Dara. Cells were stained with propidium iodide (PI) and % lysis was determined based on PI-positive cells using flow cytometry after 2 hours. (B) Levels of complement-inhibitory proteins CD46, CD55, and CD59 on the surface of Daudi or PEL cell lines were measured by flow cytometry after staining the cells with PerCP-Cy5.5-conjugated anti-CD46 or anti-CD55 antibodies or FITC-conjugated anti-CD59 antibody. (C) Levels of CD46, CD55, and CD59 on PEL cell lines expressed as fold changes over Daudi from histograms in (B).

**Supplemental Figure 3. CD38 expression and growth of ATRA-treated PEL cell lines.** (A) BC-2 and JSC-1 cell lines were treated with 0 to 40 nM ATRA for 96 hours and levels of surface CD38 were measured by flow cytometry using PerCP-Cy5.5 conjugated anti-CD38 antibody. Data shows CD38 levels expressed as median fluorescent intensity (MFI). (B) Levels of surface CD38 on BC-2, JSC-1, BCBL-1, BC-1, and BC-3 cell lines treated with DMSO (0 nM ATRA) or indicated concentrations of ATRA for 72 hours as measured by flow cytometry using PerCP-Cy5.5 conjugated anti-CD38 antibody. (C) PEL cell lines were treated for 72 hours with DMSO control or 10 nM ATRA and then live cell number was counted after trypan blue-staining. Live cell number in ATRA-treated cells is presented as a percentage of DMSO-treated cells.

**Supplemental Figure 4. ATRA does not change the levels of complement-inhibitory proteins in PEL cell lines.** Levels of complement-inhibitory proteins CD46, CD55, and CD59 on the surface of BC-2 and JSC-1 cell lines were measured 72 hours after treatment with DMSO (0 nM ATRA) or indicated concentrations of ATRA by flow cytometry after staining the cells with PerCP-Cy5.5-conjugated anti-CD46 or anti-CD55 antibodies or FITC-conjugated anti-CD59 antibody.

**Supplemental Figure 5. PBMC-mediated ADCC of BC-2 and JSC-1 cell lines.** BC-2 and JSC-1 cells were treated with DMSO control or ATRA (10 nM) or Pom (1μM for BC-2 and 0.5μM for JSC-1) for 3 days prior to measuring PBMC-mediated ADCC using calcein-AM release assay. Data is presented as lysis induced by 0.1 and 1 μg/mL Dara as a percent of that by 0 Dara. (A and B) Lysis of DMSO control-treated BC-2 (A) and JSC-1 (B) cells induced by 0, 0.1, or 1 μg/mL Dara. (C and D) Lysis of DMSO, ATRA, or Pom-treated BC-2 (C) or JSC-1 (D)

cells induced by 0.1 or 1 μg/mL Dara. Data represents averages and standard deviations from 4 separate experiments. Statistically significant difference (*P≤0.05) between 0 and 1 μg/mL ATRA as calculated using 2-tailed t-test is indicated.

**Supplemental Figure 6. Levels of CD59 in pomalidomide-treated PEL cell lines.** BC-2 and JSC-1 cell lines were treated with DMSO (0 Pom) or indicated concentrations of Pom for 72 hours and levels of surface CD59 were measured by flow cytometry using FITC-conjugated anti-CD59 antibody. (B) shows histograms from one representative experiment. (C) shows median fluorescent intensity (MFI) for CD59 on DMSO and Pom-treated cells expressed as average from 3 separate experiments. Error bars represent standard deviations.

**Supplemental Figure 7. Level of CD20 in ATRA and pomalidomide-treated PEL cell lines.** PEL cell lines BCBL-1 and BC-3 were treated for 72 hours with indicated concentrations of ATRA, pomalidomide, or DMSO control, and surface level of CD20 was measured by flow cytometry using PerCP-Cy5.5 conjugated anti-CD20 antibody. (A) Level of CD20 in BCBL-1 and BC-3 cells after 10 nM and 100 nM ATRA treatment. (B) Level of CD20 in BCBL-1 and BC-3 cells after 1 μM or 10 μM pomalidomide treatment.

**Supplemental Figure 8. CD38 levels on PEL cells from patients treated with Dara.** Flow cytometric analysis showing the phenotypic profiles of PEL cells (red) isolated from patients 1 and 2 prior to starting Dara-treatment. PEL cells were identified using a panel of antibodies as described in materials and methods. CD38, CD45, and CD19 levels on PEL cells from cerebrospinal fluid (CSF) (A) and peritoneal fluid (B) of patient 1, and from CSF of patient 2 (C)

are shown. The median MFIs for CD38 in the patients were 10,647 (CSF) and 28,078 (peritoneal fluid) for patient 1, and 33,265 (CSF) for patient 2.

## Supplemental Tables

Supplemental Table 1. Characteristics and history of patient 1 prior to Dara treatment

| | | |
|---|---|---|
| **Age at PEL diagnosis** | 34 | |
| **Sex/Gender** | Male/Cis | |
| **HIV status** | + | |
| **EBV status** | + | |
| **KSHV disease history** | | |
| Disease | MCD (2016) | |
| Treatment | Anthracycline-containing combination chemotherapy plus rituximab | |
| Outcome | Remission | |
| **Initial PEL diagnosis (2017)** | | |
| PEL location | Systemic PEL involving abdominal cavity | |
| Treatments | **Treatment** | **Outcome** |
| | 8 cycles of anthracycline-containing combination chemotherapy and splenectomy | Refractory PEL |
| | One cycle of platinum-containing combination chemotherapy | Progressive PEL with ECOG status 4 and patient transferred to NCI |
| **PEL management at NCI (2019)** | | |
| PEL location | Extensive abdominal cavity PEL with leptomeningeal involvement | |
| Prior treatments | **Treatment** | **Outcome** |
| | 200 mg pembrolizumab every 3 weeks, 4 mg pomalidomide daily 21 out of 28 days, and intrathecal methotrexate | Partial response of systemic PEL but persistent leptomeningeal disease |

Supplemental Table 2. Characteristics and history of patient 2 prior to Dara treatment

| | | |
|---|---|---|
| **Age at PEL diagnosis** | 50 | |
| **Sex/Gender** | Male/Cis | |
| **HIV status** | + | |
| **EBV status** | + | |
| **KSHV disease history** | | |
| Disease | KS and MCD (2004) | |
| Treatment | Liposomal doxorubicin and rituximab | |
| Outcome | Complete remission | |
| **PEL diagnosis (2017)** | | |
| PEL location | Extracavitary PEL with asymptomatic leptomeningeal involvement | |
| Prior treatments | **Treatment** | **Outcome** |
| | Combination anthracycline-containing chemotherapy and intrathecal methotrexate, cytarabine, and hydrocortisone | Remission of systemic PEL with refractory leptomeningeal PEL |
| | Temozolomide, etoposide, liposomal doxorubicin, and dexamethasone | Refractory leptomeningeal PEL |
| | Single agent pembrolizumab | Refractory leptomeningeal PEL |
| | Single agent pomalidomide | Refractory leptomeningeal PEL |
| | High-dose intravenous methotrexate, cytarabine, and thiotepa | Refractory leptomeningeal PEL |

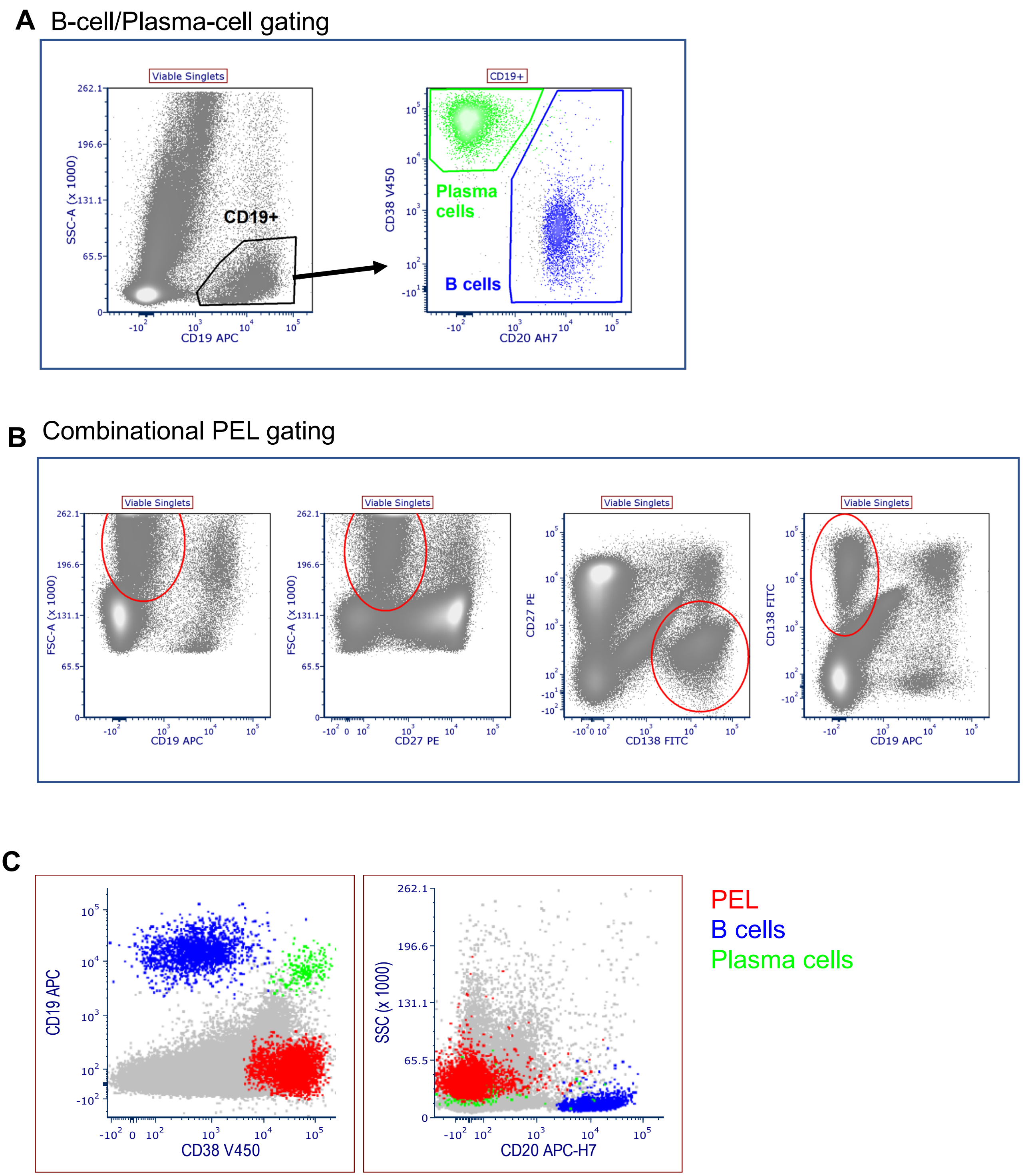


**Supplemental Figure 1**

**A**

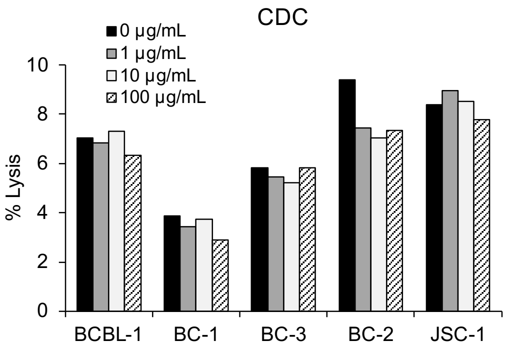


**B**

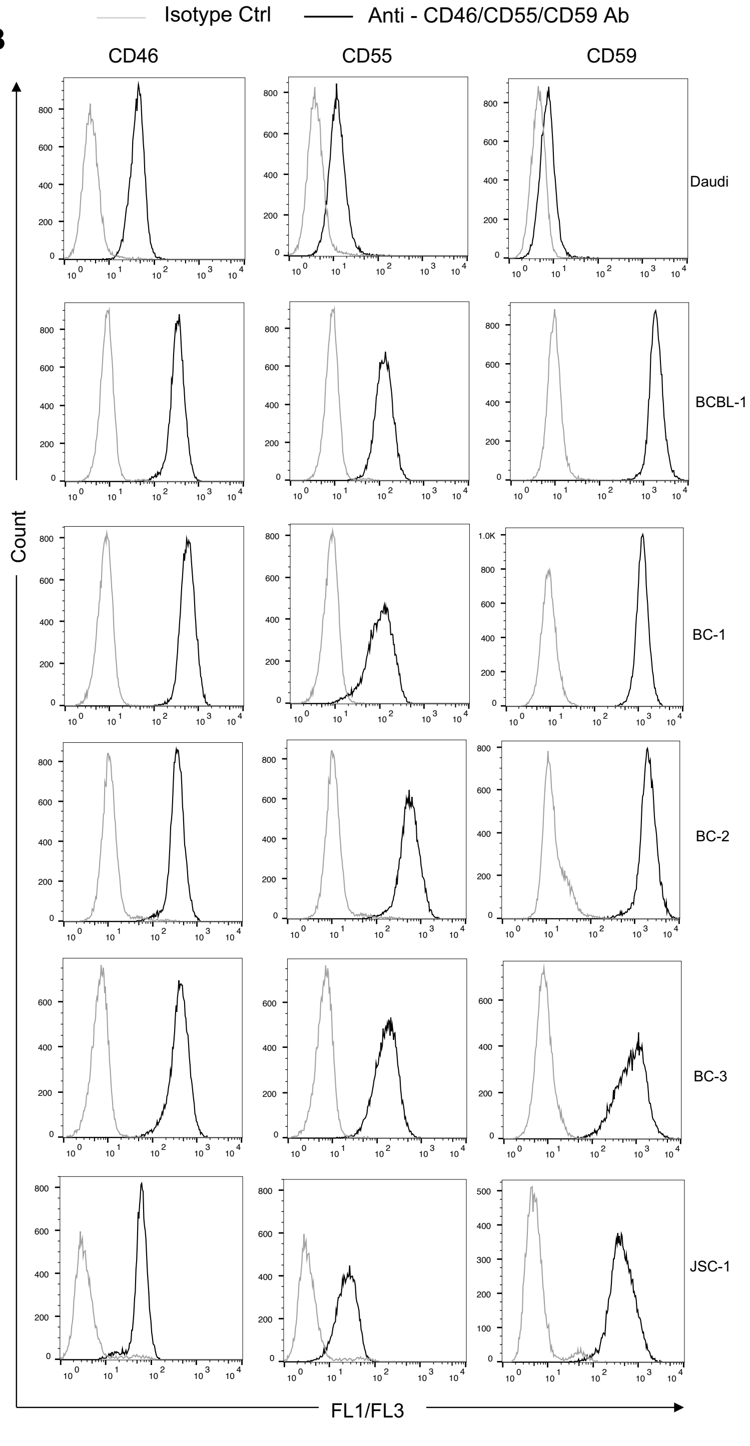


**C**

| | CD46 | CD55 | CD59 |
|---|---|---|---|
| Daudi | 1 | 1 | 1 |
| BCBL-1 | 5.8 | 9.2 | 435.8 |
| BC-1 | 10.1 | 8.0 | 282.9 |
| BC-2 | 3.3 | 22.2 | 275.7 |
| BC-3 | 7.2 | 12.3 | 181.9 |
| JSC-1 | 1.4 | 2.0 | 97.5 |

**Supplemental Figure 2**

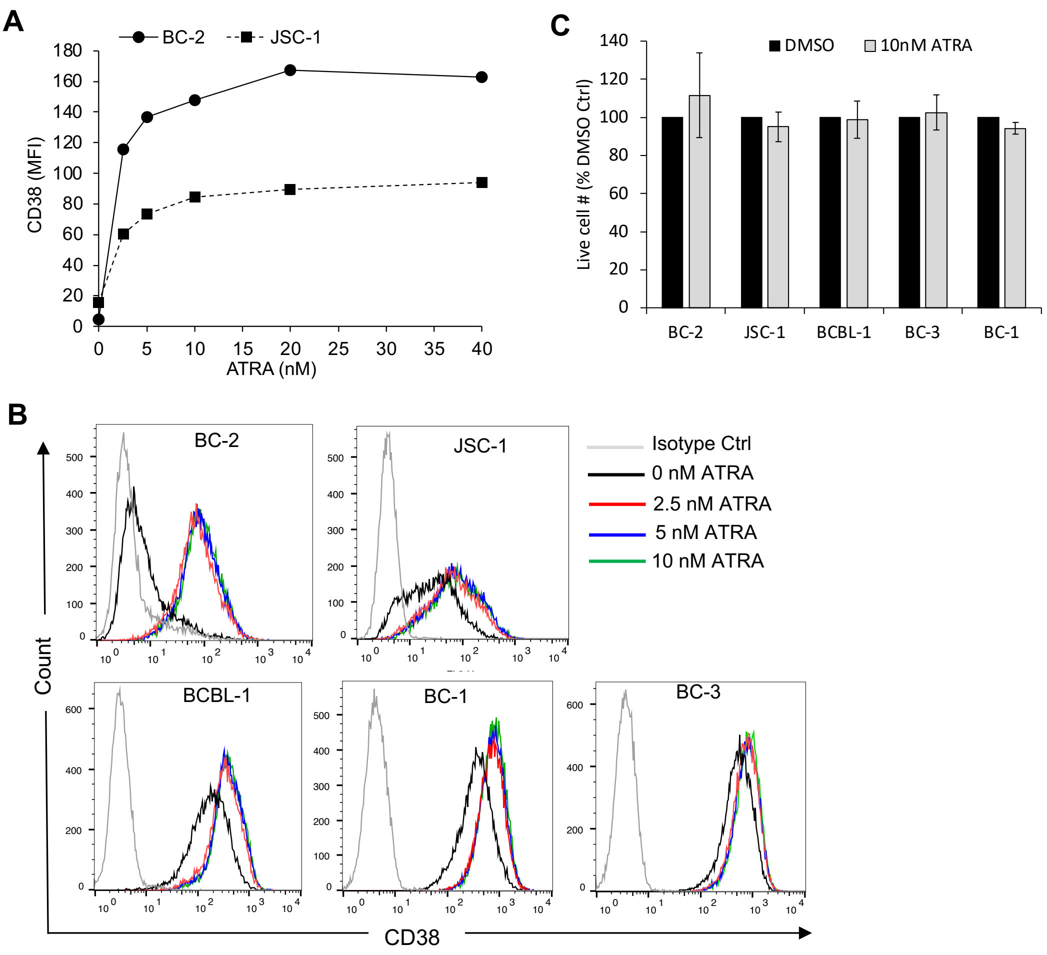


**Supplemental Figure 3**

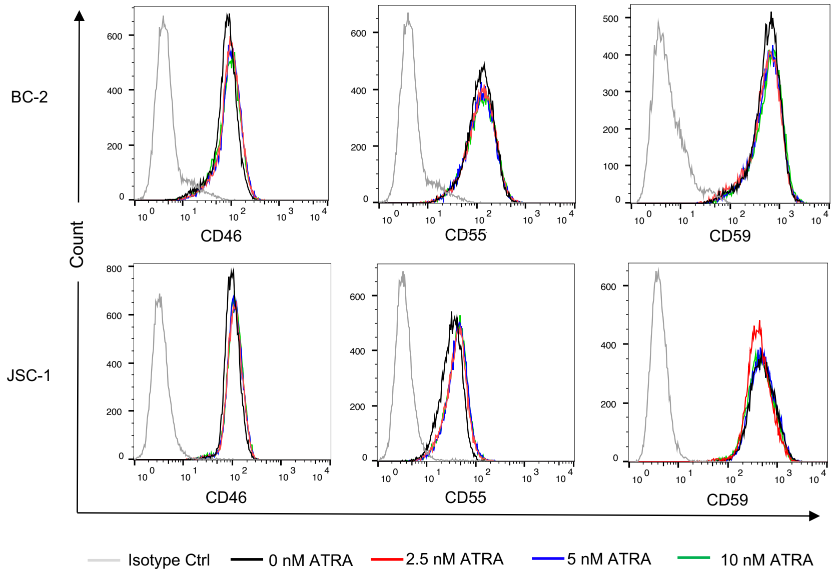


**Supplemental Figure 4**

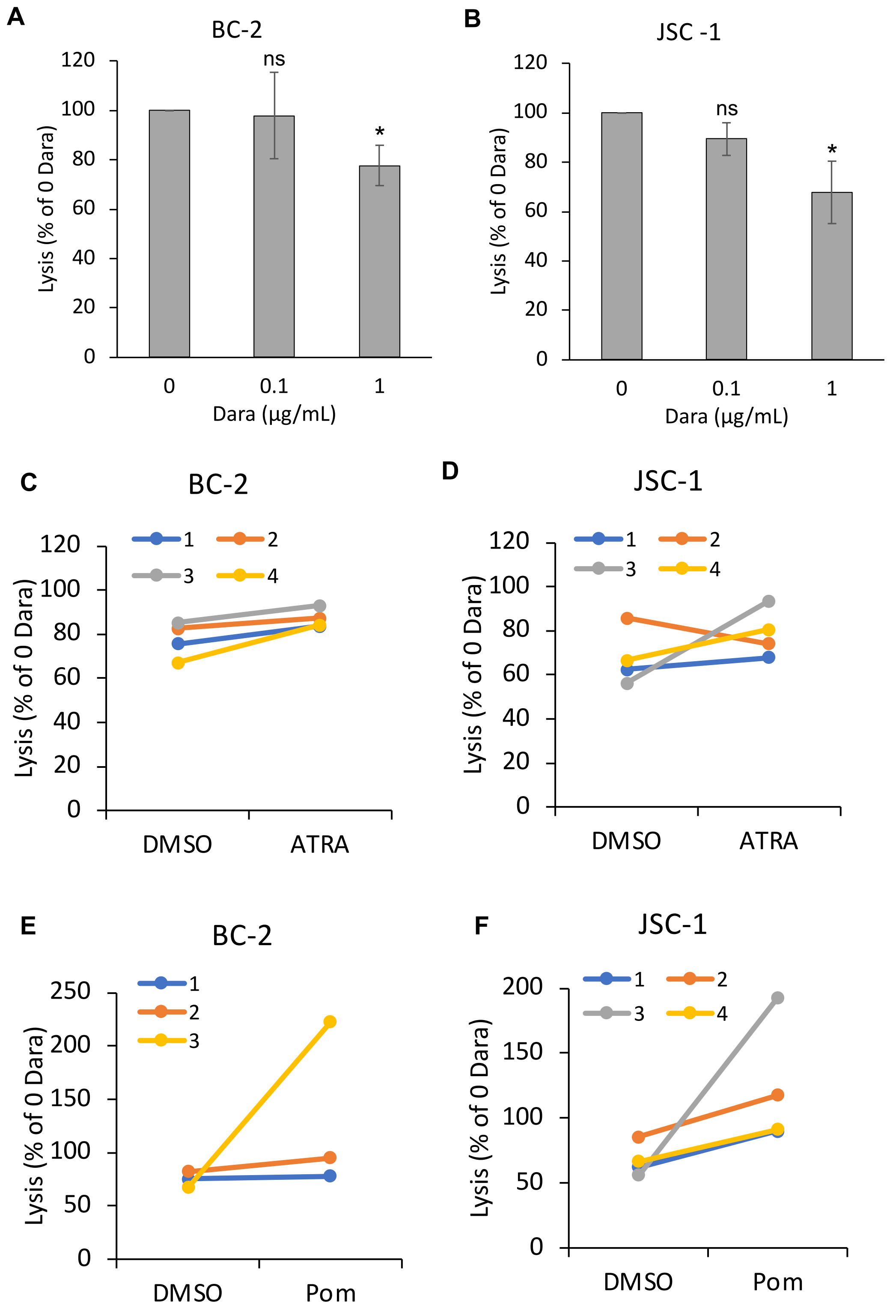


**Supplemental Figure 5**

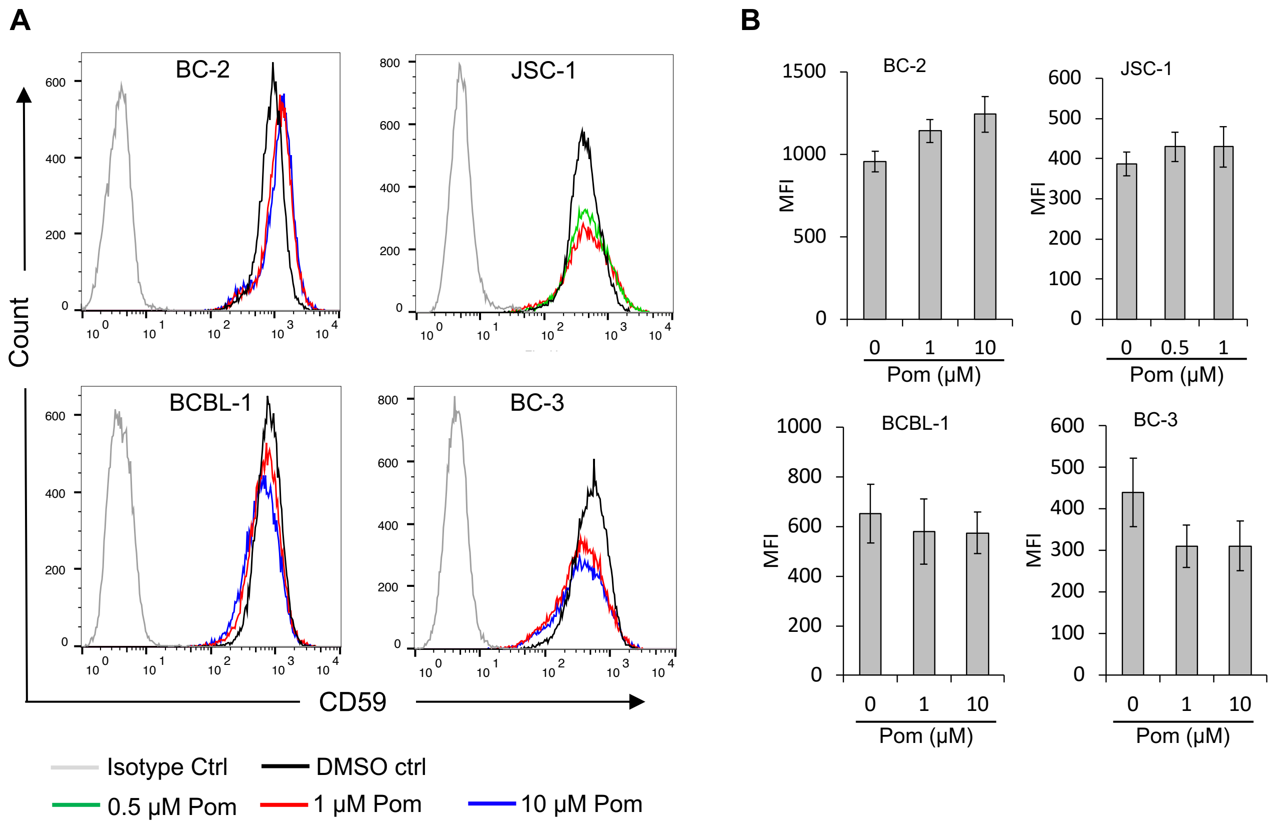


**Supplementary Figure 6**

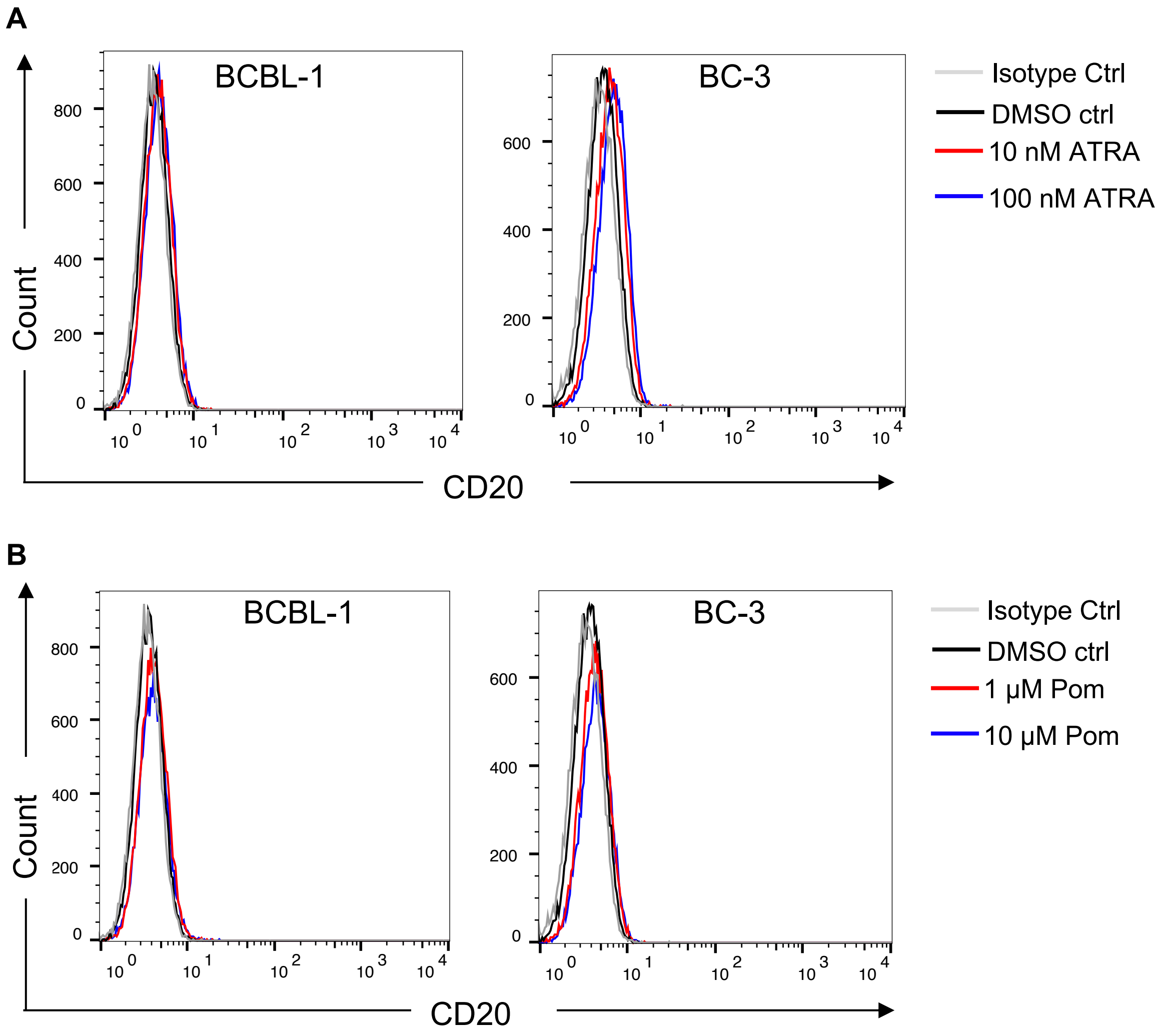


**Supplemental Figure 7**

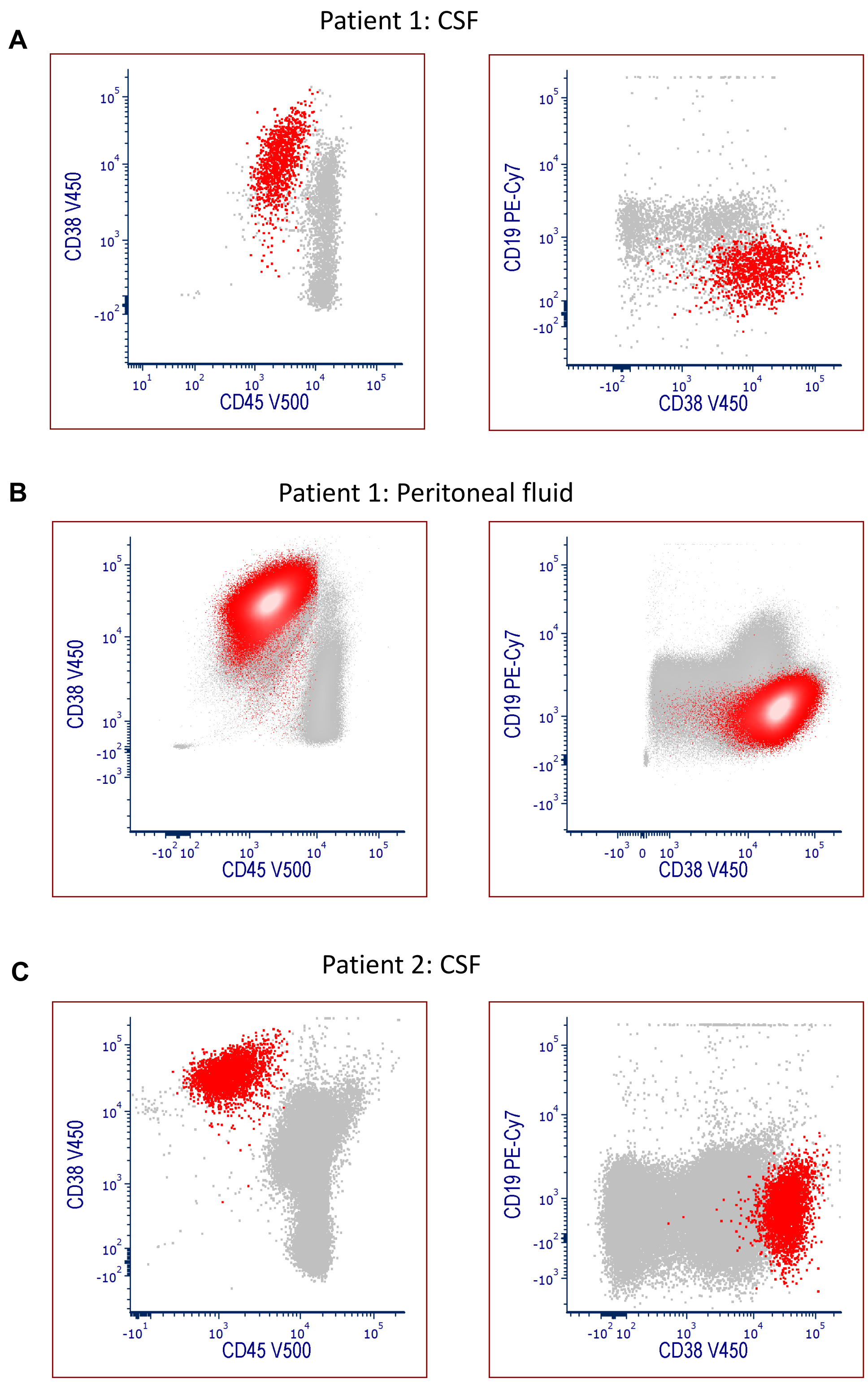


**Supplemental Figure 8**
